# Identification of Pneumococcal Serotypes by PCR–Restriction Fragment Length Polymorphism

**DOI:** 10.3390/diagnostics9040196

**Published:** 2019-11-18

**Authors:** María del Mar García-Suárez, Irene González-Rodríguez, María Dolores Cima-Cabal, Jose Enrique Yuste, Fernando Vazquez, Enrique Santiago

**Affiliations:** 1 Escuela Superior de Ingeniería y Tecnología (ESIT), Universidad Internacional de La Rioja (UNIR), 26006 Logroño, Spain; 2 Instituto de Productos Lácteos de Asturias (IPLA), 33300 Villaviciosa, Spain; 3 Centro Nacional de Microbiología, Instituto de Salud Carlos III, 28220 Madrid, Spain; 4 CIBER de Enfermedades Respiratorias, CIBERES, 28029 Madrid, Spain; 5 Servicio de Microbiología, Hospital Universitario Central de Asturias, 33011 Oviedo, Spain; 6 Área de Microbiología, Departamento de Biología Funcional, Universidad de Oviedo, 33006 Oviedo, Spain; 7 Fundación de Investigación Oftalmológica, Instituto Oftalmológico Fernández-Vega, 33012 Oviedo, Spain; 8 Instituto de Investigación Sanitaria del Principado de Asturias (ISPA), 33011 Oviedo, Spain; 9 Departamento de Biología Funcional, Universidad de Oviedo, 33006 Oviedo, Spain

**Keywords:** *Streptococcus pneumoniae*, serotype, PCR-RFLP

## Abstract

*Streptococcus pneumoniae* shows more than 90 capsular serotypes that can be distinguished by their reactivity against antisera. The main objective of this work was the development of a molecular method for serotyping without the use of antisera. A computer program containing an algorithm was used to search in a database for potentially useful enzymes for Restriction Fragment Length Polymorphism-RFLP typing, in order to maximize the discrimination between different serotypes. DNA sequences of 90 serotypes for the region between *dex*B and *ali*A genes were compiled, and a computer screening of restriction enzymes was performed. The *wzg–wzh–wzd–wze* region and *Sse*9I restriction predicted unique PCR-RFLP patterns for 39 serotypes and eight serogroups. A second restriction enzyme resolved fragment specific patterns for 25 serotypes. The method was tested with 98 serotype-unknown clinical isolates. PCR-RFLP analysis deduced correct serotypes that were confirmed by Quellung reaction for 78.5% of the isolates.

## 1. Introduction

*Streptococcus pneumoniae* produces infections such as meningitis, pneumonia, otitis or septicemia, which cause high mortality and morbidity rates in children and adults around the world. In children under 5 years of age, it is estimated that pneumococcus produces at least 3.7 million episodes of severe infections, accounting for up to 0.5 million deaths per year [1,2]. *S. pneumoniae* colonizes asymptomatically the nasopharynx of the majority of the population, especially in healthy children. From this reservoir, the infections and transmission between individuals occurs.

Evaluation of prevalent pneumococcal colonizing serotypes in the population is important because they are the main source of infections. In addition, since the introduction of the first conjugate vaccine (PCV-7) in 2000, two significant events were observed. First, a reduction in the number of invasive pneumococcal disease (IPD) by vaccine serotypes. Second, a replacement of serotypes causing IPD with the appearance of serotypes not included in the vaccine [3,4]. Serotype “switching” occurred in some cases by genetic recombination in the capsular locus, allowing the pneumococcus to escape the immunity acquired by the vaccines, which can have important consequences in the future [5,6].

The capsule is the principal line of defense against non-specific host immunity and the main virulence factor of *S. pneumoniae.* Chemical and antigenic variability of the capsule allows for the classifying of the pneumococcal population in 49 groups and more than 90 different serotypes [7,8]. The genes for the pneumococcal capsular polysaccharides (CPS) are located at the same chromosomal locus (*cps*)—between *dexB* and *aliA* [9]. The capsular polysaccharides of the most prevalent serotypes were used in the generation of the first polysaccharide vaccine (PPV-23), containing 23 different CPS, and later in the commercialization of conjugate vaccines 7, 10, or 13-valent conjugate vaccines (PCV-7, -10, -13) [1,10]. PCV-7 targeted serotypes 4, 6B, 9V, 14, 18C, 19F, and 23F. PCV-10 included also serotypes 1, 5, 7F, and, in PCV-13, serotypes 3, 6A and 19A were also added.

Detecting all the serotypes present in the nasopharynx is the key to making a good epidemiological surveillance to evaluate the impact of vaccines in the carrier state of the population. So far, the researchers serotyped isolated colonies of *S. pneumoniae* previously grown on blood agar plates, with limited identification for culture-negative samples. Molecular techniques have allowed to detect an increase in individuals colonized by more than one serotype. Additionally, other authors have revealed a multitude of genetic variations and the presence of capsular genes in other species [11]. Given the diversity of techniques used so far, WHO has given a series of recommendations to improve pneumococcal serotypes detection [12].

The gold standard for pneumococcal serotyping is the Quellung reaction [13]. This method is laborious because it requires several steps with expensive antisera. Latex agglutination is an alternative, and a cheaper option than Quellung reaction, but also requires culture of the isolates. Alternative typing methods not based in antisera are described for *S. pneumoniae* using DNA molecular methods: multiplex PCR [14,15,16], real-time PCR, microchip, sequencing-typing, reverse-hybridization, MALDI-TOF MS, and whole genome sequencing [8,17,18,19].

In this study, we report a PCR-RFLP method capable of amplifying a relatively small fragment located in the capsule locus. A statistical algorithm was used to select the best set of enzymes to discriminate the different serotypes. We have found that the digestion of the amplicons with one or two restriction enzymes yields unique patterns for 63 serotypes of *S. pneumoniae*.

## 2. Materials and Methods

### 2.1. In Silico Evaluation of Restriction Enzymes

We used an adaptation of an informatic tool [20] which analyses the efficiency of all the combinations of pairs of restriction enzymes among a set of 193 type-II restriction enzymes (Table S1) from the Rebase database [21], to specifically detect the differences between 90 DNA sequences of *S. pneumoniae* serotypes for the region defined by *wzg–wzh–wzd–wze* genes from the GenBank databases. Data generated were useful to calculate the sizes of the digestion fragments of each *S. pneumoniae* serotype and for each restriction enzyme or pair of enzymes. Briefly, the program defines the *S. pneumoniae* serotypes depending on the pattern of fragment sizes. The different *S. pneumoniae* serotypes share the same pattern if all the fragment sizes of one type are present in the other type and vice versa. The program takes into account the resolution of the electrophoresis and marks a minimum size of fragments that can be easily observed in the gels. In addition, the program analyzed the fragments produced by the restriction enzymes, investigating the enzymes in pairs.

The statistical score of a restriction enzyme for the discrimination between serotypes is calculated from a *m* × 90 table, where *m* is the number of different patterns generated by the enzyme and 90 is the number of serotypes. Ideally, the maximum number of different patterns should be 90. Ones are included in cells corresponding to each serotype (j) and its particular pattern (i). The other cells are filled with zeroes. At the end, cells including the number one in the table connect the serotypes to their patterns. The number n_ij_ in cell at row i and column j is the number of types which share the particular pattern i, and the same serotype, j. A Chi-square (χ2) value is calculated from this contingency table using the equation:

$$\chi 2=\overset{m}{\underset{i=1}{\sum }}\overset{90}{\underset{j=1}{\sum }}\frac{{\left(n_{\mathrm{ij}}-e_{\mathrm{ij}}\right)}^{2}}{e_{\mathrm{ij}}}$$

where e_ij_ = $\frac{r_{i}c_{i}}{90}\;,\;r_{i\;}=\overset{90}{\underset{j=1}{\sum }}n_{ij\;}\;\mathrm{and}\;c_{j\;}=\overset{m}{\underset{i=1}{\sum }}n_{ij\;}$ where n_ij_ is either 1 or 0.

### 2.2. Pneumococcal Isolates

Pneumococcal collection type strains were obtained from CCUG (Culture Collection University of Göteborg, Göteborg, Sweden) and Sanger Institute (Cambridgeshire, UK). Clinical isolates (98) were obtained from the Microbiology Department, Hospital Universitario Central de Asturias (Oviedo, Spain), the Spanish Pneumococcal Reference Laboratory (SPRL), National Center for Microbiology (Madrid, Spain) and University of Adelaide (Adelaide, Australia).

### 2.3. PCR Amplification of S. pneumoniae cps Genes

For PCR amplification, bacterial cells were harvested from a fresh overnight culture on blood agar plates, and a dense suspension of cells was dissolved in 100 µL of ddH_2_O. Multiplex PCR amplifications were carried out with the primers GHDE-F-7m (0.5 µM), GE-R-32m (0.8 µM), SER3-F-1 (0.1 µM), SER3-R-108 (0.1 µM), GE-R-34, GLF-F-151, and GE-R-38 (Table S4). The reactions mix was carried out in a volume of 50 µL, with 0.5 units of HotMaster Taq Polymerase (5PRIME, VWR International Eurolab, Barcelona, Spain), buffer 1×, MgCl_2_ 1.5 mM, dNTPs 10 mM each, and 5 µL of cell suspensions. PCR was undertaken in a T-personal Thermocycler (Biometra, Göttingen, Germany) with initial denaturation at 94 °C for 5 s, followed by 30 cycles of 94 °C for 5 s, 48 °C for 20 s and 65 °C for 10 min, and a final extension at 65 °C for 10 min.

### 2.4. Analysis of Fragments Obtained from Enzyme Digestions

Ten microliters of PCR products were digested with different restriction enzymes (*Sse*9I, *Alu*I, *Mse*I, *Bfu*CI, *Bst*DEI, *Esa*BC3I, *Hpy*CH4IV) in an appropriate restriction buffer to a total volume of 50 µL. After incubation for 3 h at the recommended temperature, the digested DNA was electrophoresed on a 2.5% non-denaturing polyacrylamide gel with 1.5% of Spreadex polymer (Elchrom Scientific, Cham, Switzerland), and the sizes of the fragments were estimated by comparison with 20-bp ladder (Lonza Rockland, Inc, Rockland, ME, USA).

### 2.5. Serotyping of Clinical Isolates

Conventional serotyping was performed by the Quellung reaction and a dot blot assay, using rabbit polyclonal antisera from the Statens Serum Institute, Copenhagen, Denmark, as previously described [22], at SPRL, Madrid, Spain.

## 3. Results

### 3.1. In Silico Analysis of GenBank Sequences

**Figure 1:**
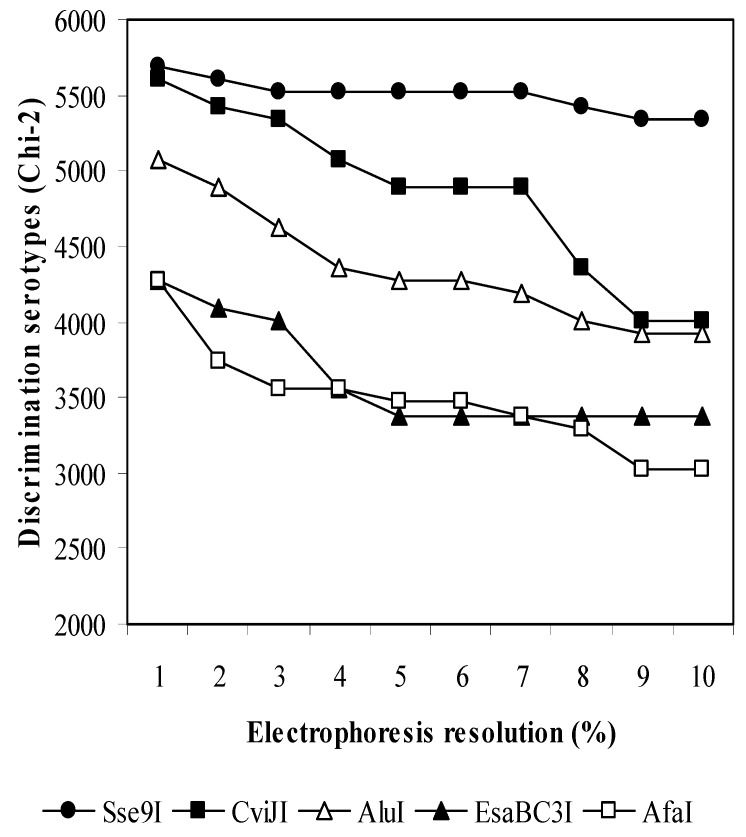
Discrimination values between serotypes by analysis of fragments produced by digestions with different restriction enzymes in the region *wzg–wzh–wzd–wze*. The values are given as a function of the resolution of the electrophoresis system. All the fragments of 100 bp and over are considered for the analysis.

The genes *wzg–wzh–wzd–wze*, located in the locus *cps*, were tested by a computer screening of restriction enzymes. Virtually, the resolution of the electrophoresis system could be maximum, 0% (when the fragments with different lengths are always identified as different fragments), or minimum, 10% (when two fragments differing by less than 10% are considered to be the same fragment). Serotype discrimination reaches higher levels when the fragments generated by the enzymes differ the most from each other. Ideally, for 90 serotypes the number of different fragment patterns should be 90, but we cannot obtain this number by testing either single or double enzyme digestions. Two restriction enzymes, *Sse*9I and *Cvi*J, showed better scores of discrimination among serotypes (Figure 1) than the rest of enzymes, since they generated 60 and 55 different patterns, respectively, at an electrophoresis resolution of 1%.

### 3.2. Generation of a Preliminary Database of Fragment Patterns.

Sequences from GenBank corresponding to the region *wzg–wzh–wzd–wze* were tested by informatic analysis with *Sse*9I (…↓AATT…) (NIPPON Genetics Europe, Germany) and the fragment patterns obtained generated the initial database with different pattern fragments (Table S2a). In this database, sequences belonging to 39 serotypes (1, 3, 4, 5, 6A, 6B, 7C, 8, 10A, 10B, 11F, 12B, 14, 15A, 15F, 18A, 18C, 19A, 19B, 19C, 19F, 21, 23A, 23B, 23F, 24A, 24F, 27, 31, 33B, 33C, 33D, 34, 37, 41F, 43, 45, 47A, and 48) showed unique fragment patterns. In six cases we also have detected two fragment patterns for the same serotype (6A, 6B, 8, 18C, 23F and 24A, see patterns nº 7 and 8, 9 and 10, 42 and 43). Sixteen serotypes showed the same pattern as another serotype within same serogroup (see patterns nº 11, 15, 16, 19, 21, 28, 39 and 49). Sequences belonging to 37 serotypes showed the same pattern as another serotypes (see patterns nº 2, 12, 20, 23, 25, 30, 31, 47, 50, 56).

For discrimination of serotypes without a unique fragment pattern, the informatic tool was used to predict the optimal secondary restriction enzymes. The results obtained showed that the following enzymes could be used to discriminate: 7A and 7F: *Bfu*CI; 9L and 9N: *Bst*DEI, *Cvi*AII, *Hinf*I; 11B and 11C: *Mse*I; 13 and 20: *Mse*I, *Bst*4CI; 25A and 25F: *Hpy*CH4IV; 28A and 28F: *Mse*I*, Alu*I*, Bst*4CI, *Esa*BC3I; 29 and 39: *Mse*I*, Hpy*CH4IV, *Esa*BC3I; 33D and 41F: *Mse*I*, Hpy*CH4IV, *Esa*BC3I; 35F and 47F: *Esa*BC3I. A second restriction enzyme resolved a fragment-specific pattern for sequences belonging to 25 serotypes: 7A, 7F, 9L, 9N, 11B, 11C, 12F, 13, 16A, 17A, 17F, 20, 25A, 25F, 28A, 28F, 29, 33D, 35F, 36, 39, 41F, 42, 44, and 47F (Table S3). However, due to similarity of sequences, serotypes 2/41A, 7B/40, 9A/9V, 10C/10F, 11A/11D/18F, 12A/46, 15B/15C, 18B/18C, 22A/22F, 32A/32F and 33A/33C/33F/35A/35B/35C could not be discriminated using the PCR-RFLP method.

### 3.3. PCR-RFLP Analysis of S. pneumoniae Collection Type Strains

To validate the database of fragment patterns, 37 collection type strains of *S. pneumoniae* (Table 1) were tested by PCR-RFLP analysis. First, we tried to amplify all serotypes with the primers GHDE-F-7m and GE-R-32m, but repeatedly, no amplification products were found for some strains. In order to amplify all serotypes of *S. pneumoniae*, PCR primers were designed to include specifically the serotypes 3, 25, 29, 38, 39 and 43 (Table S4, Figure S1). PCR was performed in suspensions of colonies and PCR products were obtained from all strains (Figure S2). As expected, PCR-RFLP from collection type strains belonged to the following serotypes 1, 2, 3, 4, 5, 6B, 9A, 10B, 10F, 11A, 11B, 11D, 12A, 12B, 14, 18C, 19A, 19F, 20, 21, 24F, 27, 31, 32A, 33D, 33F, 35A, 40, 42, 45 and 48, which showed patterns already included in the initial fragment database. Surprisingly, the reference strains with serotypes 7A, 10C, 19C, 22F, 34 and 36, showed fragment patterns distinct to those calculated based on the sequence from the database. These patterns were identified (Table S2b) and added to the initial database.

### 3.4. Serotype Identification of Clinical Isolates by PCR-RFLP Analysis

**Figure 2:**
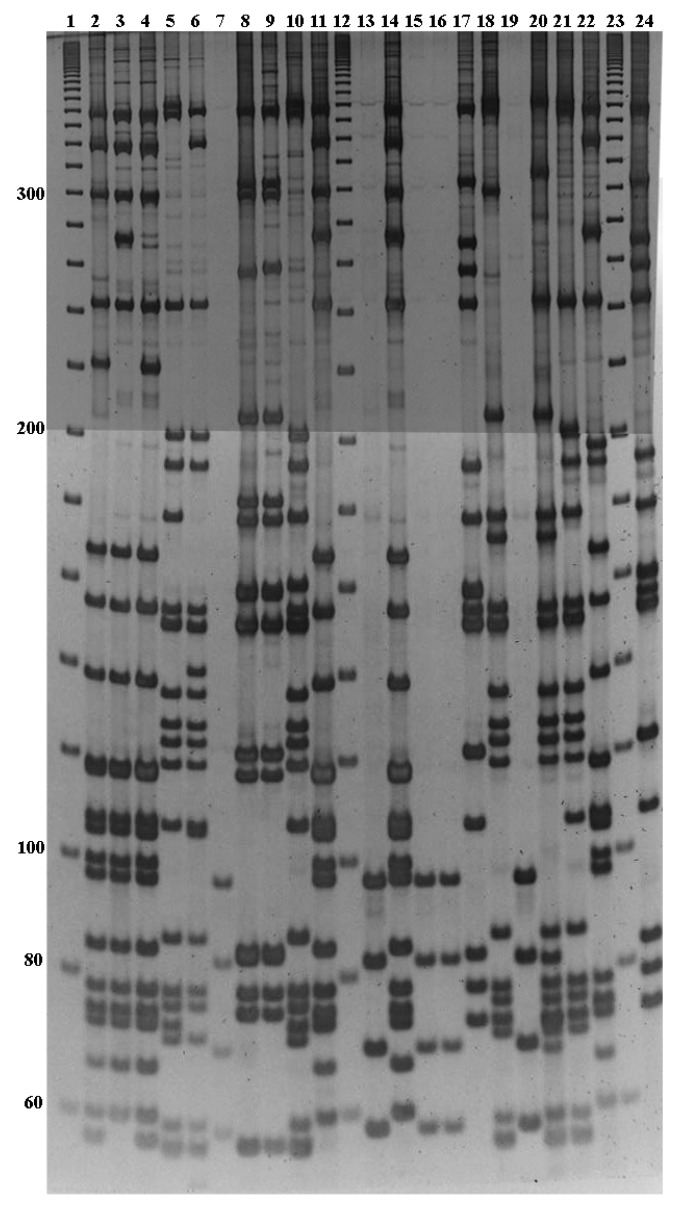
Identification of serotypes from *Sse*9I fragments in polyacrylamide gels stained with SYBR Green I. Lane 1, 20 bp ladder; lane 2, serotype 14; lane 3, serotype 7A/7F; lane 4, serotype 14; lane 5, serotype 22F; lane 6, serotype 8; lane 7, serotype 3; lane 8, serotype 6B; lane 9, serotype 6B; lane10, serotype 17A/17F/33A/33F/35/42; lane11, serotype 7A/7F; lane12, 20 bp ladder; lane 13, serotype 3; lane14, serotype 7A/7F; lane 15, serotype 3; lane 16, serotype 3; lane 17, serotype 11A/11D/18F; lane 18, serotype 28A/28F; lane 19, serotype 3; lane 20, serotype 12F; lane 21, serotype 22F; lane 22, serotype 16F; lane 23, 20 bp ladder; lane 24, serotype 11A/11D/18F.

In order to check the potential of the PCR-RFLP method, 98 clinical isolates with an unknown serotype were tested in a blinded fashion. Amplification was obtained for all isolates, except one (95.5%). PCR-RFLP with *Sse*9I from some clinical isolates are shown in Figure 2. The fragment patterns were easily obtained and compared with the database to obtain the corresponding serotype. A total of 77 isolates (78.5%) were serotyped correctly (i.e., in agreement with Quellung reaction results) including 26 serotypes (73.2%): 1 (*n* = 3), 2 (*n* = 1), 3 (*n* = 8), 4 (*n* = 4), 7F (*n* = 7), 8 (*n* = 4), 9N (*n* = 2), 9V (*n* = 3), 10A (*n* = 1), 11A (*n* = 4), 12F (*n* = 2), 14 (*n* = 6), 15A (*n* = 1), 16F (*n* = 2), 19A (*n* = 3), 19F (*n* = 1), 22F (*n* = 3), 23B (*n* = 2), 23F (*n* = 7), 24F (*n* = 1), 28A (*n* = 1), 29 (*n* = 1), 33F (*n* = 2), 34 (*n* = 2), 35B (*n* = 2), 35F (*n* = 2) (Table 2). Additionally, 21 isolates revealed 15 new PCR-RFLP fragment patterns, corresponding to serotypes 6A, 6B, 7F, 12F, 13, 14, 15A, 15B, 17F, 18C, 19A and 24F (Table S2c). As expected, for the two non-typeable isolates no amplification was achieved. Clinical isolates had been obtained from different geographical regions in Spain (except serotype 7F and 8, from University of Adelaide) containing the 32 serotypes: 1, 2, 3, 4, 6A, 6B, 7F, 8, 9N, 9V, 10A, 11A, 12F, 13, 14, 15A, 15B, 16F, 17F, 18C, 19A, 19F, 22F, 23B, 23F, 24F, 28A, 29, 33F, 34, 35B, 35F. Serotyping of the strains was done at the Spanish Pneumococcal Reference Laboratory, using Quellung reaction.

## 4. Discussion

In this report, we describe a molecular method to identify the 63 serotypes and eight serogroups of *S. pneumoniae* without the use of antiserum. We have attempted to develop an approach to serotyping using the same PCR primers for all the serotypes and an RFLP step for resolution of the serotype/serogroup. In the PCR-RFLP approach, the selection of sequences within the *cps* gene cluster is critical, as well as the particular set of restriction enzymes used to generate fragments. A computer program previously developed to discriminate HPV types [20] was modified to search for the optimal restriction enzyme for discriminating the 90 *S. pneumoniae* serotypes. The selection of the sequences *wzg*–*wzh*–*wzd*–*wze* allowed to obtain PCR amplifications with an acceptable size to facilitate positive amplifications in all the strains. It also allowed easy interpretation of agarose gels and database. Previous reports used for PCR-RFLP large fragments as the region between *cps*A-*cps*B and the region between *dex*B-*ali*A [23,24,25]. On the other hand, several methods, using different sizes of amplicons to differentiate serotypes, have been described [14,15,26,27].

Although Quellung reaction has been considered the gold standard technique for pneumococcal serotyping [28,29,30], this technique is time-consuming, and the need for a diverse panel of specific antisera makes the test very expensive in order to identify the group, type and factor. These problems might be solved by using molecular methods such as real-time PCR, multiplex-PCR, capsular sequence typing, or whole genome sequencing [31,32,33,34,35].

There are many other advantages in using the presented method to type pneumococci. The choice of *Taq* polymerase and the purification of high-quality DNA were not critical aspects for good quality patterns, since amplification products were obtained using the PCR primers described in this report, which are smaller than *dex*B-*ali*A amplifications previously reported. Furthermore, direct amplification from cell suspensions simplifies the routine assays and reduces time-consuming DNA purification protocols. Multiplex PCR with a reduced number of primers provides a cost-effective method. Furthermore, the serotypes tested in the current study, included those approved by the FDA regarding the heptavalent pneumococcal conjugate vaccine PCV7, PCV10, PCV13, and phase III clinical trials with a fifteen-valent PCV [36], and all of the 23 serotypes in the PPV23 vaccine.

After the introduction of pneumococcal conjugate vaccines, an increase in specific serotypes causing IPD in both children and adults occurred. In Spain, pneumococcal vaccines became available for private purchase in 2001 (PCV7), 2009 (PCV10) and 2010 (PCV13). Overall, non-PCV13 serotypes have raised in recent years, contributing to the current burden of IPD cases in children and adults [4,37]. Moreover, high colonization rates have been observed in Spanish children, with serotypes 19A, 16F and 15B being the most prevalent [38]. Other serotypes, such as 19A, 3 and 6A, remain important contributors to IPD after PCV13. In Europe, the most prevalent serotypes causing community-acquired pneumonia in adults are included in PCV13, and bacteraemic pneumonia is mainly caused by serotypes 1, 3, 7F, 19A and 14 [39,40]. The PCR-RFLP system described here may be used to easily discriminate the prevalent serotypes 1, 3, 6A, 14 and 19A, included in PCV13, as well as non-PCV13 serotypes such as 24F, 23B and 10A.

Due to similarity of sequences, serotypes 2/41A, 7B/40, 9A/9V, 10C/10F, 11A/11D/18F, 12A/46, 15B/15C, 18B/18C, 22A/22F, 32A/32F and 33A/33C/33F/35A/35B/35C could not be discriminated using the PCR-RFLP method described and should be monitored using specific serotype primers, previously described (http://www.cdc.gov/streplab/pcr.html), or sequencing. One of the limitations of this paper is that it does not include serotypes 6C, 6D, and some genetic variants described [41,42,43].

Furthermore, the appearance of new patterns reveals the constant change of capsular genes, especially among serotypes exposed to the pressure of immunization, and the diversity within pneumococcal *cps* locus sequences, as previously described [5]. The database should always remain open to include changes that will take place in the future with the implementation of new vaccines.

In summary, the PCR-RFLP analysis of the *cps* genes with *Sse*9I digestion proved to be an adequate tool for the correct serotyping of almost all prevalent serotypes and serogroups of *S. pneumoniae*. This method needs few PCR primers and one or two restriction enzymes, making it technically assumable by most research laboratories and pneumococcal reference laboratories.

## Figures and Tables

**Table 1:** Pneumococcal collection type strains used in the study.

Strain
*S. pneumoniae* serotype 1 CCUG 2839A ^a^
*S. pneumoniae* serotype 2 CCUG 8435 ^a^
*S. pneumoniae* serotype 3 GB05 ^b^
*S. pneumoniae* serotype 4 CCUG 2226 ^a^
*S. pneumoniae* serotype 5 CCUG 2541 ^a^
*S. pneumoniae* serotype 6B CCUG 1350 ^a^
*S. pneumoniae* serotype 7A CCUG 8436 ^a^
*S. pneumoniae* serotype 9A CCUG 3506 ^a^
*S. pneumoniae* serotype 10B Sri Lanka ^b^
*S. pneumoniae* serotype 10C/1 Sanger ^b^
*S. pneumoniae* serotype 10F CCUG 5697 ^a^
*S. pneumoniae* serotype 11A CCUG 36617 ^a^
*S. pneumoniae* serotype 11B CCUG 8440 ^a^
*S. pneumoniae* serotype 11D Sanger 70/86 ^b^
*S. pneumoniae* serotype 12A CCUG 8444 ^a^
*S. pneumoniae* serotype 12B Gambia 1/81 ^b^
*S. pneumoniae* serotype 14 CCUG 1086B ^a^
*S. pneumoniae* serotype 18C Sanger 4593/4 ^b^
*S. pneumoniae* serotype 19A Sanger 1773/39 ^b^
*S. pneumoniae* serotype 19C Sanger 408/41 ^b^
*S. pneumoniae* serotype 19F CCUG 1407 ^a^
*S. pneumoniae* serotype 20 CCUG 8451 ^a^
*S. pneumoniae* serotype 21 CCUG 1697 ^a^
*S. pneumoniae* serotype 22F Sanger 1772/40 ^b^
*S. pneumoniae* serotype 24F CCUG 8457 ^a^
*S. pneumoniae* serotype 27 CCUG 5898 ^a^
*S. pneumoniae* serotype 31 CCUG 6956 ^a^
*S. pneumoniae* serotype 32A CCUG 8458 ^a^
*S. pneumoniae* serotype 33D India ^b^
*S. pneumoniae* serotype 33F Sanger 3077/37 ^b^
*S. pneumoniae* serotype 34 CCUG 2399 ^a^
*S. pneumoniae* serotype 35A CCUG 3556 ^a^
*S. pneumoniae* serotype 36 CCUG 5906 ^a^
*S. pneumoniae* serotype 40 CCUG 8468 ^a^
*S. pneumoniae* serotype 42 CCUG 6568 ^a^
*S. pneumoniae* serotype 45 CCUG 8472 ^a^
*S. pneumoniae* serotype 48 CCUG 8476 ^a^

^a^ CCUG, Culture Collection University of Göteborg, and ^b^ Sanger Institute (Cambridgeshire, UK).

**Table 2:** Summary of PCR-RFLP analysis of clinical isolates.

Serotype	Nº Isolates	Origin	Nº Concordant Patterns (nº Strains)	Nº New Patterns (nº Strains)
1	3	^a^SPRL	3 (3)	
2	1	^b^HUCA	1 (1)	
3	8	SPRL	8 (8)	
4	4	HUCA, SPRL	4 (4)	
6A	1	HUCA		1 (1)
6B	3	HUCA		2 (3)
7F	8	^c^UA, SPRL	7 (7)	1(1)
8	4	UA, SPRL	4 (4)	
9N	2	HUCA, SPRL	2 (2)	
9V	3	HUCA, SPRL	3 (3)	
10A	1	SPRL	1 (1)	
11A	4	HUCA, SPRL	4 (4)	
12F	3	^d^CNM, SPRL	2 (2)	1 (1)
13	1	HUCA		1(1)
14	7	HUCA, SPRL	6 (6)	1 (1)
15A	2	SPRL	1 (1)	1 (1)
15B	1	CNM		1 (1)
16F	2	SPRL	2 (2)	
17F	1	CNM		1 (1)
18C	1	SPRL		1 (1)
19A	11	SPRL	3 (3)	3 (8)
19F	1	HUCA	1 (1)	
22F	3	HUCA, SPRL	3 (3)	
23B	2	SPRL	2 (2)	
23F	7	HUCA, SPRL	7 (7)	
24F	2	SPRL	1 (1)	1 (1)
28A	1	SPRL	1 (1)	
29	1	SPRL	1 (1)	
33F	2	SPRL	2 (2)	
34	2	HUCA, SPRL	2 (2)	
35B	2	SPRL	2 (2)	
35F	2	HUCA, SPRL	2 (2)	
NT	2	SPRL	2 (2)	
Total	98		77 (77)	15 (21)

^a^ Clinical isolates from Spanish Pneumococcal Reference Laboratory, SPRL (nº strains): serotype 1 (3), serotype 3 (9), serotype 4 (3), serotype 7F (8), serotype 8 (3), serotype 9N (1), serotype 10A (1), serotype 11 (3), serotype 12F (2), serotype 14(6), serotype 15A (2), serotype 16F (2), serotype 18C (1), serotype 19A (11), serotype 22F(2), serotype 23B (2), serotype 23F (1), serotype 24F (2), serotype 28 (1), serotype 29 (1), serotype 33F (2), serotype 34 (1), serotype 35B (2), serotype 35F (1), NT, non-typeable (2). ^b^ Clinical isolates from Hospital Universitario Central de Asturias, HUCA: *S. pneumoniae* serotype 2 (strain 99/18413), serotype 4 (strain 08/2751), serotype 6A (strain 99/23686), serotype 6B (strain 99/34320), serotype 6B (strain 08/2720), serotype 6B (strain 99/16243), serotype 9N (strain 00/14800), serotype 9V (strain 99/39253), serotype 9V (strain 00/10762), serotype 11A (strain 99/22279), serotype 13 (strain 99/34157), serotype 14 (strain 00/37682), serotype 19F (strain 00/8339), serotype 22F (strain 99/29369), serotype 23F (strain 00/42913), serotype 23F (strain 1655 Badalona), serotype 23F (strain 99/17077), serotype 23F (strain 99/30466), serotype 23F (strain 08/2721), serotype 34 (strain 00/3495), serotype 35F (strain 00/1522). ^c^ Clinical isolates from University of Adelaide: *S. pneumoniae* serotype 7F UA, *S. pneumoniae* serotype 8 UA. ^d^ Clinical isolates from Centro Nacional de Microbiología, CNM: *S. pneumoniae* serotype 9V CNM, serotype 10A CNM, serotype 12F CNM, serotype 15B CNM, serotype 17F CNM.

## Supplementary Materials

The following are available online at https://www.mdpi.com/2075-4418/9/4/196/s1, Table S1: Restriction enzymes considered for discrimination among 90 *S. pneumoniae* serotypes, Table S2a: *Sse9I* fragment sizes obtained by analysis of GenBank sequences of the 90 *S. pneumoniae* serotypes. Table S2b: New patterns obtained by PCR-RFLP analysis of reference strains. Table S2c: New patterns obtained by PCR-RFLP analysis of clinical isolates. Table S3: Patterns obtained with secondary restriction enzymes. Table S4: Oligonucleotide primers used for multiplex PCR in this study. Figure S1. Location of specific primers for serotypes 3, 25, 38, 29, 39 and 43 in *cps* locus. Figure S2. PCR products obtained from clinical isolates.
