# Supplementary material for: Identification of Pneumococcal Serotypes by PCR–Restriction Fragment Length Polymorphism
Source: Diagnostics (Basel). 2019 Nov 18;9(4):196. doi: 10.3390/diagnostics9040196 (PMC6963424; doi:10.3390/diagnostics9040196)
Supplement: Supplementary file 1 [file diagnostics-09-00196-s001.zip › diagnostics-632678 suppl for final/Figure S2.pdf]

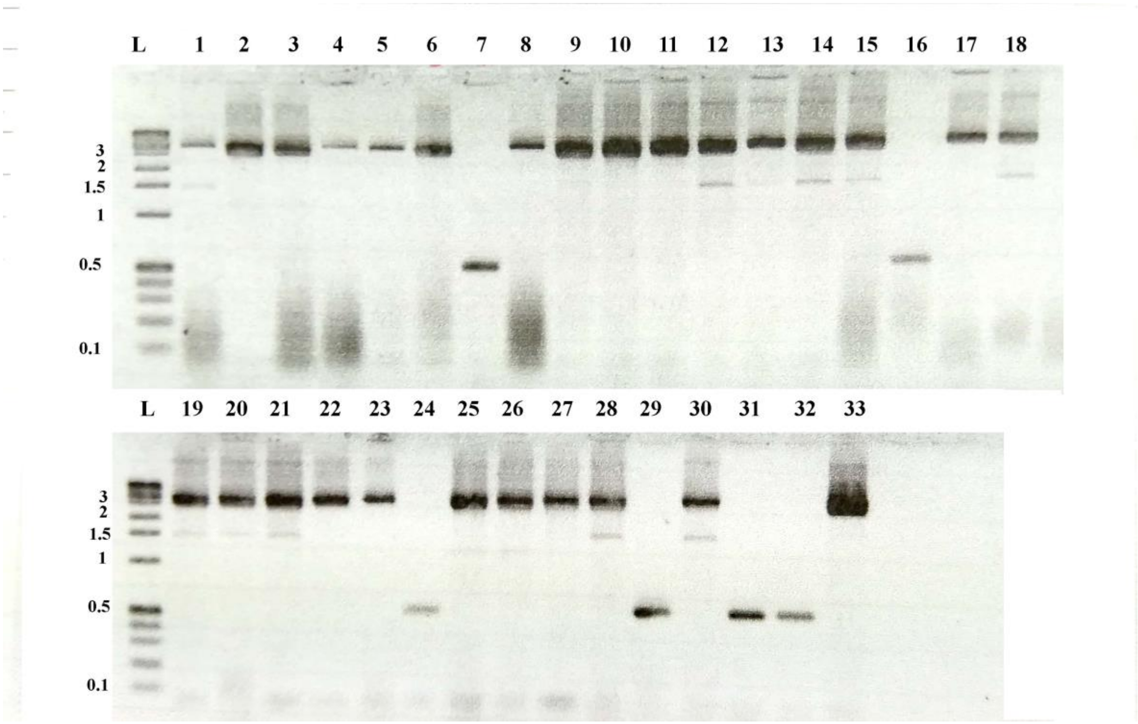

Figure S2. PCR products obtained from clinical isolates. L, Perfect marker DNA 0.1-12Kbp (Novagen); lane 1: serotype 7F; lane 2: serotype 4; lane 3: serotype 19A; lane 4: serotype 7F; lane 5: serotype 23B; lane 6: serotype 33F; lane 7: serotype 3; lane 8: serotype 4; lane 9: serotype 29; lane 10: serotype 9N; lane 11: serotype 18C; lane 12: serotype 7F; lane 13: serotype 1; lane 14: serotype 14; lane 15: serotype 14; lane 16: serotype 3; lane 17: serotype 19A; lane 18: serotype 7F; lane 19: serotype 14; lane 20: serotype 7F; lane 21: serotype 14; lane 22: serotype 22F; lane 23: serotype 8; lane 24: serotype 3; lane 25: serotype 19A; lane 26; serotype 19A; lane 27: serotype 35B; lane 28: serotype 7F; lane 29: serotype 3; lane 30: serotype 7F; lane 31: serotype 3; lane 32: serotype 3; lane 33: serotype 11.
