# Supplementary material for: Identification of Pneumococcal Serotypes by PCR–Restriction Fragment Length Polymorphism
Source: Diagnostics (Basel). 2019 Nov 18;9(4):196. doi: 10.3390/diagnostics9040196 (PMC6963424; doi:10.3390/diagnostics9040196)
Supplement: Supplementary file 1 [file diagnostics-09-00196-s001.zip › diagnostics-632678 suppl for final/Table S1.pdf]

**Table S1.** Restriction enzymes considered for discrimination among 90 *S. pneumoniae* serotypes.

| | | | | | | | | |
|---|---|---|---|---|---|---|---|---|
| *Aat*I | *Age*I | *Asp*S9I | *Bis*I | *Bsi*WI | *Bst*X2I | *Gdi*II | *Pac*I | *Ssp*I |
| *Aat*II | *Aha*III | *Ass*I | *Blp*I | *Bsp*19I | *Cci*NI | *Hae*I | *Pae*R7I | *Tat*I |
| *Acc*I | *Ahd*I | *Asu*II | *Bme*1390I | *Bsp*68I | *Cdi*I | *Hgi*EII | *Pas*I | *Tau*I |
| *Acc*II | *Ahl*I | *Asu*C2I | *Bme*1580I | *Bsp*143II | *Cfr*I | *Hin*4II | *Pfe*I | *Tsp*RI |
| *Acc*III | *Aji*I | *Asu*NHI | *Bmg*I | *Bsp*1286I | *Cfr*9I | *Hinc*II | *Pfl*1108I | *Tss*I |
| *Acc*16I | *Ajn*I | *Ava*II | *Bmi*I | *Bsp*1407I | *Cfr*42I | *Hind*III | *Pfo*I | *su*I |
| *Acc*65I | *Ale*I | *Ava*III | *Box*I | *Bsp*GI | *Cje*NII | *Hinf*I | *Ppu*MI | *Uba*F11I |
| *Acc*B1I | *Alu*I | *Axy*I | *Bpu*10I | *Bsp*HI | *Cju*I | *Hpa*I | *Psi*I | *Uba*PI |
| *Acc*B7I | *Alw*21I | *Bal*I | *Bpv*UI | *Bsp*LU11I | *Cju*II | *Hpy*8I | *Psp*XI | *Xba*I |
| *Acc*BSI | *Alw*44I | *Bam*HI | *Bsa*AI | *Bsp*MAI | *Cpo*I | *Hpy*99I | *Pvu*II | |
| *Aci*I | *Alw*NI | *Ban*II | *Bsa*BI | *Bsp*NCI | *Cvi*AII | *Hpy*178III | *Sal*I | |
| *Acl*I | *Ama*87I | *Ban*III | *Bsa*JI | *Bss*NAI | *Cvi*JI | *Hpy*188I | *San*DI | |
| *Acl*WI | *Apa*I | *Bau*I | *Bsa*MI | *Bss*T1I | *Cvi*RI | *Hpy*CH4IV | *Sbf*I | |
| *Aco*I | *Apa*BI | *Bbe*I | *Bsb*I | *Bst*4CI | *Dra*II | *Mab*I | *Sfi*I | |
| *Acs*I | *Ape*KI | *Bbu*I | *Bsc*GI | *Bst*C8I | *Drd*II | *Mae*III | *Sgr*AI | |
| *Acv*I | *Asc*I | *Bbv*CI | *Bse*1I | *Bst*DEI | *Ecl*136II | *Mfe*I | *Sgr*DI | |
| *Acy*I | *Ase*I | *Bcl*I | *Bse*118I | *Bst*DSI | *Eco*32I | *Mlu*I | *Sim*I | |
| *Ade*I | *Asi*SI | *Bet*I | *Bse*PI | *Bst*EII | *Eco*RI | *Mro*NI | *Smi*I | |
| *Afa*I | *Asp*I | *Bfa*I | *Bse*X3I | *Bst*ENI | *Esa*BC3I | *Mse*I | *Sml*I | |
| *Afe*I | *Asp*700I | *Bfm*I | *Bse*YI | *Bst*MWI | *Fau*NDI | *Msl*I | *Srf*I | |
| *Afi*I | *Asp*A2I | *Bfu*CI | *Bsh*1285I | *Bst*NSI | *Fin*I | *Msp*A1I | *Sse*9I | |
| *Afl*II | *Asp*CNI | *Bgl*I | *Bsh*FI | *Bst*SNI | *Fse*I | *Mss*I | *Sse*232I | |
| *Afl*III | *Asp*LEI | *Bgl*II | *Bsi*SI | *Bst*XI | *Fsp*AI | *Nmu*CI | *Sse*8647I | |
