# Supplementary material for: Identification of Pneumococcal Serotypes by PCR–Restriction Fragment Length Polymorphism
Source: Diagnostics (Basel). 2019 Nov 18;9(4):196. doi: 10.3390/diagnostics9040196 (PMC6963424; doi:10.3390/diagnostics9040196)
Supplement: Supplementary file 1 [file diagnostics-09-00196-s001.zip › diagnostics-632678 suppl for final/Table S2a.pdf]

Table S2a. *Sse*9I fragment sizes obtained by analysis of GenBank sequences of the 90 *S. pneumoniae* serotypes.

[a]Pattern nº	Serotype	GenBank	*Sse*9I fragments ordered by size (bp)[b]														
1	1	CR931632	374	336	270	238	230	165	152	139	134	115	114	114	104	101	
	1	FQ312030															
2	2	CR931633	374	336	241	197	191	175	152	146	133	126	121	117	104		
	41A	CR931713															
	8	AJ239004	374	336	241	197	191	175	152	**147**	**146**	133	126	121	117	104	102
3	3	CR931634	543	306	270	254	134	120	116	115	115	114	114	104	102		
4	3	*galU-pmg*	93	78	64	54	46	43	38	35	25	17	12				
5	4	CR931635	384	295	270	259	175	174	159	152	147	146	121	115	115	114	
	4	AE005672															
	4	AF316639															
6	5	CR931637	429	385	191	179	165	159	152	134	115	114	114	104	104	101	
7	6A	JF911492	384	374	197	152	**148**	147	146	139	137	133	126	121	117	**115**	104
8	6A	LR216014	384	374	**230**	197	**191**	**175**	152	147	146	139	133	126	121	117	104
9	6B	CR931639	384	374	197	191	175	152	148	147	146	**139**	133	126	121	117	104
10	6B	AF316640	384	374	197	191	175	152	**151**	148	147	146	133	126	121	117	104
11	7A	CR931640	374	336	295	270	241	165	152	134	115	114	114	104	101		
	7F	CR931643															
12	7B	CR931641	374	336	270	241	191	165	152	134	115	114	114	104	104	101	
	40	CR931712															
	16F	CR931668	374	336	270	241	**195**	**191**	165	152	134	115	114	104	104	101	
13	7C	CR931642	384	374	270	241	191	175	165	152	147	146	115	114	104	101	
14	8	CR931644	374	336	241	197	191	152	147	146	137	133	126	121	117	104	102
	8	AF316641															
15	9A	CR931645	384	374	295	293	204	197	152	150	133	126	121	117			
	9V	CR931648															
	9V	AF402095															
16	9L	CR931646	374	336	295	270	204	165	152	134	115	114	114	101			
	9N	CR931647															

17	10A	CR931649	583	384	259	193	191	175	159	152	147	146	115	107	104		
18	10B	CR931650	583	468	384	374	295	193	159	152	107						
19	10C	CR931651	384	374	295	252	214	204	175	158	152	147	146	119	119	101	
	10F	CR931652															
20	11A	CR931653	374	304	266	252	241	191	175	158	155	152	147	146	119	119	104
	11D	CR931656															
	18F	CR931674															
21	11B	CR931654	374	304	295	266	252	175	159	158	156	155	147	146	134	119	119
	11C	CR931655															
22	11F	CR931657	384	374	293	204	197	191	175	152	133	126	121	117	114	104	
23	12A	CR931658	384	374	295	241	175	168	152	147	133	126	121	117			
	12F	CR931660															
	44	CR931717															
	46	CR931719															
	32A	CR931696	384	374	295	241	175	168	152	**147**	**146**	133	126	121	117		
	32F	CR931697															
24	12B	CR931659	384	374	238	204	175	168	152	147	133	126	121	117			
25	13	CR931661	374	306	295	270	241	165	152	134	115	114	114	101			
	20	CR931679															
26	14	CR931662	374	336	295	241	217	165	152	134	115	114	114	104	101		
	14	X85787															
	14	FQ312029															
	14	CZ693492	374	336	295	241	217	165	152	134	115	114	114	**106**	101		
27	15A	CR931663	384	374	295	230	197	175	165	158	152	147	139	101			
28	15B	CR931664	384	374	270	246	241	152	147	146	115	115	114	104	101		
	15C	CR931665															
29	15F	CR931666	384	374	295	197	175	165	158	148	147	139	134	101			
30	16A	CR931667	374	336	295	270	230	165	152	139	134	115	114	114	104		
	36	CR931708															
	18C	CR931673	374	336	295	270	230	165	**153**	**152**	139	134	114	114	104	101	
	18B	CR931672															

31	17A	CR931669	384	374	197	191	175	159	152	147	146	133	126	121	117	104	
	17F	CR931670															
	33A	CR931698															
	33F	CR931702															
	33F	AJ006986															
	35A	CR931704															
	35B	CR931705															
	35C	CR931706															
	42	CR931715															
32	18A	CR931671	374	369	306	270	191	165	152	134	115	114	114	104	104	101	
33	18C	NZ_ABAE00000000	374	304	295	252	180	175	159	158	155	147	146	119	119	114	
34	19A	CR931675	374	304	295	252	204	180	175	158	155	147	146	126	119	114	
35	19B	CR931676	374	336	295	270	246	241	152	134	115	114	114	114	104	101	
36	19C	CR931677	669	384	270	241	175	165	152	147	146	115	114	101			
37	19F	CR931678	374	336	295	230	165	152	150	139	134	120	115	114	114	104	101
	19F	AF030368															
	19F	AF30370															
	19F	AF030371															
	19F	CZ693534															
38	21	CR931680	384	374	295	204	175	152	147	146	133	126	126	121	117		
39	22A	CR931681	384	374	295	241	197	175	152	147	146	133	126	121	117		
	22F	CR931682															
40	23A	CR931683	374	336	270	191	165	159	152	146	115	114	104	104	102	101	
41	23B	CR931684	374	304	295	252	214	204	175	158	155	147	146	119	119	114	111
42	23F	CR931685	374	336	**295**	241	197	175	152	146	133	126	121	117	102		
	23F	AF057294															
	23F	CZ693502															
43	23F	AF030373	374	336	241	**238**	197	175	152	146	133	126	121	117	102		
	23F	CP026670															
44	24A	CR931686	374	336	295	270	165	152	148	146	139	115	114	104	102	101	
45	24(2236/42)	CR931687	384	374	295	270	241	165	152	147	146	115	114	104	101		

46	24F	CR931688	374	336	295	270	230	165	152	146	139	115	114	104	102	101		
47	25A	*glf-wze*	403	275	236	219	191	189	186	170	157	122	104					
	25F																	
	38																	
	25A	CR931689	389	316	282	248	224	191	159	150	140	108	104					
	25F	CR931690																
	38	CR931710																
48	27	CR931691	374	336	241	191	175	168	152	147	146	133	126	121	117	104		
49	28A	CR931692	384	374	295	204	175	168	152	147	146	133	126	121	117			
	28F	CR931693																
50	29	CR931694	389	374	336	295	282	204	152	146	140	108	102					
	39	CR931711																
51	31	CR931695	336	259	241	191	175	168	152	147	146	133	126	121	117	115	104	102
52	33B	CR931699	384	374	295	241	197	175	152	147	146	133	126	117				
53	33C	CR931700	384	374	197	191	175	159	152	147	146	133	126	121	117	104	102	
54	33D	CR931701	384	374	241	197	191	175	152	147	133	126	117	104				
55	34	CR931703	384	374	295	197	175	159	152	147	146	133	126	121	117			
56	35F	CR931707	384	374	270	191	165	159	152	147	146	115	114	114	104	101		
	47F	CR931721																
57	37	CR931709	374	336	295	197	182	175	152	146	133	126	121	117	102			
58	41F	CR931714	384	374	241	191	175	152	147	146	133	126	126	121	117	104		
59	43	CR931716	389	316	282	248	224	191	159	150	140	108	104					
60	45	CR931718	384	374	270	204	191	175	174	152	147	146	121	115	114	104	102	
61	47A	CR931720	374	336	295	241	197	175	152	134	133	126	121	117	114			
62	48	CR931722	526	295	270	243	204	165	134	115	114	114	101					

Patterns are considered different for an electrophoresis resolution of 1%.
Only fragments above 100 bp are showed, except serotype 3 (*galU-pmg*), (underlined fragments cannot be observed in the gel).
Fragments marked in green are considerate the same fragment by the software.
Fragments marked in red are distinct fragment for a same serotype.
