# Supplementary material for: Identification of Pneumococcal Serotypes by PCR–Restriction Fragment Length Polymorphism
Source: Diagnostics (Basel). 2019 Nov 18;9(4):196. doi: 10.3390/diagnostics9040196 (PMC6963424; doi:10.3390/diagnostics9040196)
Supplement: Supplementary file 1 [file diagnostics-09-00196-s001.zip › diagnostics-632678 suppl for final/Table S2b.pdf]

Table S2b. New patterns obtained by PCR-RFLP analysis of reference strains.

**Serotype**	***Sse*9I fragments ordered by size (bp)**												
7A	374	300	278	230	162	150	140	138	120	114	101		
10C	384	300	295	258	190	178	160	158	130	120			
19C	384	278	190	178	170	162	158	150	118	112			
22F	384	374	241	197	191	175	152	147	146	133	126	121	117	104
34	384	374	295	192	178	162	152	150	138	133	121	119	114	
36	374	300	240	192	165	158	152	118	100				

S. pneumoniae serotype 7A CCUG 8436, *S. pneumoniae* serotype 10C/1 Sanger, *S. pneumoniae* serotype 19C Sanger 408/41, *S. pneumoniae* serotype 22F Sanger 1772/40, *S. pneumoniae* serotype 34 CCUG 2399 and *S. pneumoniae* serotype 36 CCUG 5906.
