# Supplementary material for: Identification of Pneumococcal Serotypes by PCR–Restriction Fragment Length Polymorphism
Source: Diagnostics (Basel). 2019 Nov 18;9(4):196. doi: 10.3390/diagnostics9040196 (PMC6963424; doi:10.3390/diagnostics9040196)
Supplement: Supplementary file 1 [file diagnostics-09-00196-s001.zip › diagnostics-632678 suppl for final/Table S2c.pdf]

Table S2c. New patterns obtained by PCR-RFLP analysis of clinical isolates.

**Serotype**	***Sse*9I fragments ordered by size (bp)**														
6A	384	374	210	197	191	175	152	147	146	139	133	126	121	117	104
6B	384	374	197	191	175	152	148	147	146	139	133	126	121	117	104
7F	384	374	295	293	204	165	152	134	115	114	114	104	101		
12F	384	310	241	175	168	152	147	133	126	121	117				
12F	384	309	238	204	175	168	152	147	133	126	121	117			
13	374	310	295	270	241	165	152	134	115	114	114	100			
14	374	336	295	220	217	165	152	134	115	114	114	104	101		
15A	374	336	304	270	242	204	150	147	117	114	101				
15B	500	336	270	243	204	195	191	148	115	114	104	101			
17F	384	374	295	197	175	159	152	147	146	133	126	121	117		
18C	420	384	374	295	241	165	152	134	115	114	104	101			
19A	374	304	295	252	204	180	175	158	157	155	147	146	119	119	114
19A	374	304	295	256	204	180	175	159	147	119	114				
19A	374	304	255	241	204	180	175	159	152	147	119	114			
24F	384	374	270	241	191	165	152	147	115	114	104	103			
