# Supplementary material for: Identification of Pneumococcal Serotypes by PCR–Restriction Fragment Length Polymorphism
Source: Diagnostics (Basel). 2019 Nov 18;9(4):196. doi: 10.3390/diagnostics9040196 (PMC6963424; doi:10.3390/diagnostics9040196)
Supplement: Supplementary file 1 [file diagnostics-09-00196-s001.zip › diagnostics-632678 suppl for final/Table S3.pdf]

Table S3. Patterns obtained with secondary restriction enzymes.

Enzyme	Serotype	Digestion fragments ordered by size (bp)															
*Bfu*CI	7A	829	676	362	342	266	246	203	161	136							
	7F	829	676	362	342	266	203	200	161	136							
*Bst*DEI	9L	772	602	408	300	238	224	210	144	126	117						
	9N	772	602	399	238	224	210	209	150	150	144	117					
*Mse*I	11B	846	468	315	300	220	197	191	175	167	160						
	11C	846	468	315	300	220	197	191	175	160	129						
*Mse*I	13	543	315	222	216	206	186	167	156	147	136	120	117	114	110	103	
	20	398	315	222	216	206	186	167	156	147	145	136	120	117	114	110	103
*Mse*I	29	468	334	292	252	245	206	204	197	167	146	145	120	110			
	39	613	334	292	252	245	206	204	197	167	146	120	110				
*Alu*I	28A	582	363	256	234	226	191	188	186	183	142	141					
	28F	582	363	256	234	226	191	188	186	183	150	142	141				
*Hpy*CH4IV	25A	1007	405	223	211	201	105										
	25F	1007	405	278	223	201	105										
*Mse*I	33D	822	659	398	315	197	167	160	145	110							
	41F	822	659	543	315	197	167	145	110								
*Esa*BC3I	35F	1095	361	344	305	279	245	223	204	161							
	47F	1095	361	344	305	292	245	223	204	161							
