# Supplementary material for: Identification of Pneumococcal Serotypes by PCR–Restriction Fragment Length Polymorphism
Source: Diagnostics (Basel). 2019 Nov 18;9(4):196. doi: 10.3390/diagnostics9040196 (PMC6963424; doi:10.3390/diagnostics9040196)
Supplement: Supplementary file 1 [file diagnostics-09-00196-s001.zip › diagnostics-632678 suppl for final/Table S4.pdf]

**Table S4.** Oligonucleotide primers used for multiplex PCR in this study.

| **Primer** | **Sequence (5′ to 3′)** | **Gene** | **Genome position** | **GenBank** | **Amplicon size (bp)** |
|---|---|---|---|---|---|
| GHDE-F-7m | CACAGAAAGTGAAGCGAAGTGT | *wzg* | 320105-320126 | NC_003028 | 2000-3306 |
| GE-R-32m | AT(G/A)TA(G/A)TCAAAATATTTACGCAA(G/T)GT | *wze* | 323411-323386 | | |
| GE-R-34[a] (+GHDE-F7m) | ACATAATCAAAATACTTACGCATAGT | *wze* | 4538-4513 | CR931694 | 3306 |
| GLF-F-151[b] | TCTACTGTGAGAATATAGAAGGAGTTA | *glf* | 5920-5946 | CR931710 | 2519 |
| GE-R-38[b] | ATATAGTCAAAGTACTTTTTCATAGA | *wze* | 8349-8324 | | |
| SER3-F-1[c] | CATTGAATAAGACACAGAGTGTT | *galU* | 9221-9243 | CR931634 | 505 |
| SER3-R-108[c] | TTCAAAAGCGAATTCTGGTGAAAAAT | *pgm* | 9725-9700 | | |

[a]Primer specific to amplify serotypes 29, 39 and 43; [b]Primer specific to amplify serotypes 25 and 38; [c]Primer specific to amplify serotype 3.
